# Comparison of different prediction models for estimation of walking and running energy expenditure based on a wristwear three-axis accelerometer

**DOI:** 10.3389/fphys.2023.1202737

**Published:** 2023-10-30

**Authors:** Luyou Xu, Jinxi Zhang, Zhen Li, Yu Liu, Zhuang Jia, Xiaowei Han, Chenglin Liu, Zhixiong Zhou

**Affiliations:** ^1^ Institute for Sport Performance and Health Promotion, Capital University of Physical Education and Sports, Beijing, China; ^2^ Institute of Artificial Intelligence in Sports, Capital University of Physical Education and Sports, Beijing, China; ^3^ School of Physical Education and Sport Science, Fujian Normal University, Fuzhou, China; ^4^ Faculty of Education, Beijing Normal University, Beijing, China

**Keywords:** physical activity, METs, artificial neural network, tri-axis accelerometer, energy consumption, metabolic prediction model, predicting energy expenditure, high accuracy

## Abstract

**Objective:** Objectively and efficiently measuring physical activity is a common issue facing the fields of medicine, public health, education, and sports worldwide. In response to the problem of low accuracy in predicting energy consumption during human motion using accelerometers, a prediction model for asynchronous energy consumption in the human body is established through various algorithms, and the accuracy of the model is evaluated. The optimal energy consumption prediction model is selected to provide theoretical reference for selecting reasonable algorithms to predict energy consumption during human motion.

**Methods:** A total of 100 subjects aged 18–30 years participated in the study. Experimental data for all subjects are randomly divided into the modeling group (*n* = 70) and validation group (*n* = 30). Each participant wore a triaxial accelerometer, COSMED Quark pulmonary function tester (Quark PFT), and heart rate band at the same time, and completed the tasks of walking (speed range: 2 km/h, 3 km/h, 4 km/h, 5 km/h, and 6 km/h) and running (speed range: 7 km/h, 8 km/h, and 9 km/h) sequentially. The prediction models were built using accelerometer data as the independent variable and the metabolic equivalents (METs) as the dependent variable. To calculate the prediction accuracy of the models, root mean square error (RMSE) and bias were used, and the consistency of each prediction model was evaluated based on Bland–Altman analysis.

**Results:** The linear equation, logarithmic equation, cubic equation, artificial neural network (ANN) model, and walking-and-running two-stage model were established. According to the validation results, our proposed walking-and-running two-stage model showed the smallest overall EE prediction error (RMSE = 0.76 METs, Bias = 0.02 METs) and the best performance in Bland–Altman analysis. Additionally, it had the lowest error in predicting EE during walking (RMSE = 0.66 METs, Bias = 0.03 METs) and running (RMSE = 0.90 METs, Bias < 0.01 METs) separately, as well as high accuracy in predicting EE at each single speed.

**Conclusion:** The ANN-based walking-and-running two-stage model established by separating walking and running can better estimate the walking and running EE, the improvement of energy consumption prediction accuracy will be conducive to more accurate to monitor the energy consumption of PA.

## 1 Introduction

Physical activity (PA) deficiency has become the fourth leading cause of death in the world. About 5 million people die of PA deficiency every year (WHO, 2020). Positive PA is closely related to health; for example, moderate physical activity (MPA) is inversely proportional to the occurrence of depression (Dishman et al., 2021). Proper PA can help reduce the risk of abnormal blood lipids, improve the levels of cholesterol and high-density lipoprotein cholesterol, and promote the development of blood indicators in a healthy direction (Delavar et al., 2011; Montesi et al., 2013). There is a dose–effect relationship between PA and health. Wen et al. found that 90 min of MPA per week (or 15 min per day) can significantly reduce the risk of death related to all causes, cancer, cardiovascular disease, and diabetes (Wen et al., 2011). The WHO recommends that adults engage in MPA for more than 30 min at least 5 times a week, or vigorous physical activity (VPA) for more than 20 min at least 3 times a week (Bull et al., 2020).

Objective measurement^1^ of PA is an essential basis for monitoring whether one has reached the recommended levels. Currently, wearable devices based on triaxial accelerometers are the primary means of objectively measuring PA. According to the 2023 Global Fitness Trend Survey Report by the American Sports Medical Association, wearable technology is ranked first. However, numerous studies have shown that wearable devices are ineffective in predicting EE to meet the needs of consumers. A meta-analysis of 158 articles, which included Apple, Fitbit, Garmin, Mio, Misfit, Polar, Samsung, Withings, and Xiaomi commercial wearable devices, demonstrated that none of the products can effectively predict EE (Fuller et al., 2020).

The accuracy of estimating EE using three-axis accelerometers primarily depends on the establishment of a prediction model based on the relationship between three-axis acceleration data of the accelerometer and METs. This involves calculating the various intensities of physical activity and then determining EE using the model. Building a prediction model between acceleration data and METs is crucial to the accuracy of the estimation. Currently, there are many models used to predict physical activity based on triaxial accelerometers. Initially, the most widely established models were linear equations, with the linear equations developed by Freedson being the most classic. The equation is based on z-axis acceleration: *M* *E* *T* *s* = 1.439008 + 0.000795 × *A* *C* *z*, which is widely accepted among scholars (Freedson et al., 1998).

As research progresses, the nonlinear equations developed by Campbell et al. has been increasingly utilized in the creation of EE prediction models. This equation employs a multiple stepwise regression method to analyze various factors and establish the nonlinear equation $EEact\;\left(k\right)=aN\times \left(k\right)1+bN\times V\left(k\right)p2$ , where a and b, and p1 and p2 represent the coefficients associated with height and weight, respectively (Campbell et al., 2002). Couter et al. proposed the establishment of subsection equations based on linear or nonlinear equations to enhance the accuracy of EE monitoring. The findings indicate that the difference between the measured and predicted values of the subsection equations is significantly smaller compared to other types of equations (Crouter et al., 2006).

With the development of AI, machine learning, as a subset of AI, has received extensive attention in recent years. Taking the features extracted from acceleration data as the input variables, through a series of machine-learning algorithms, such as the ANN model (Mackintosh et al., 2016), random forest (Ellis et al., 2014), support vector machine (Liu et al., 2011) etc., the functional relationship with the output variables is obtained (i.e., the machine-learning model is generated). Mackintosh established an ANN model through the characteristic values of the mean and variance of the accelerometer within 15 s and evaluated the model. The results showed that there was no significant difference between the predicted value and measured value, and that the accuracy of the MET prediction was better than that of Freedson’s linear EE prediction equation for children (Mackintosh et al., 2016). Ellis established a regression forest based on the wrist accelerometer and proved that it effectively improved the accuracy of the model prediction (Ellis et al., 2014).

This study was aimed at the problem of the dependence of the accuracy of the three-axis accelerometer in the estimation of the EE of human motion on the built prediction model. Using acceleration data collected from wrist accelerometers, we constructed multiple prediction models for EE during walking and running and evaluated their accuracies. Based on the evaluation, we selected the superior model to provide a theoretical reference for choosing an appropriate EE prediction model for walking and running. At the same time, it is conducive to promoting the scientific development of sports.

## 2 Materials and methods

### 2.1 Participants

A total of 100 students aged 18–30, including 50 men and 50 women. Experimental data for all participants were recruited and randomly assigned to either the modeling group (*n* = 70) or the validation group (*n* = 30) while maintaining gender balance in each group. The gender, height, weight, BMI, and body fat ratio of the participants are shown in Table 1. All participants completed a Physical Activity Readiness Questionnaire (PAR-Q) and pre-exercise health screening questionnaire and provided signed informed consent. Inclusion criteria required no restrictions on sports participation and the ability to complete the physical activities of the experimental tests. All of the procedures in this study were performed in accordance with the Declaration of Helsinki. The study was approved by the Review Committee of the Capital University of Physical Education and Sports.

**TABLE 1:** Characteristics of participants (mean ± SD).

	Total (*n* = 100)	Modeling (*n* = 70)	Validation (*n* = 30)
Age (years)	24.4 ± 1.6	24.2 ± 1.6	24.7 ± 1.6
Height (m)	1.7 ± 0.08	1.7 ± 0.08	1.7 ± 0.09
Body mass (kg)	64.0 ± 10.6	64.0 ± 10.2	64.8 ± 11.6
BMI (kg/m^2^)	22.0 ± 2.1	22.0 ± 2.1	22.1 ± 2.2
Body fat rate (%)	21.6 ± 6.5	21.5 ± 6.7	22.0 ± 6.2

Note: SD, standard deviation; m, meter; kg, kilogram; BMI, body mass index.

### 2.2 Experimental design

**FIGURE 1:**
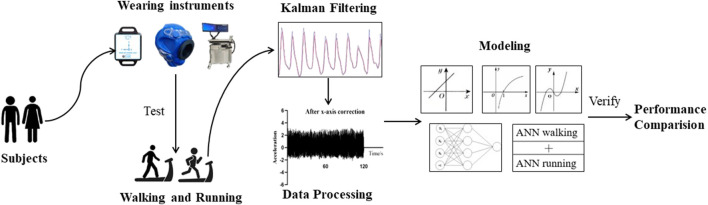
Research structure chart.

Prior to the experiment, participants were asked to refrain from strenuous exercise for 24 h, and avoid consuming any food or drink containing caffeine. The experiment was conducted 1.5–2 h after a meal. Upon arrival at the laboratory, participants rested for at least 10 min, during which their height, weight, and body composition were measured. Triaxial accelerometers, a QUARK PFT, and heart rate monitors were attached to participants for the sitting, walking, and running experiments. For the walking experiment, participants walked on a treadmill at speeds of 2 km/h, 3 km/h, 4 km/h, 5 km/h, and 6 km/h, with a uniform step frequency and natural arm swing. For the running experiment, the participants ran on the treadmill at speeds of 7 km/h, 8 km/h, and 9 km/h, with a uniform step frequency and arms bent at the elbows and clenched fists while naturally swinging their arms back and forth. The walking and running tests were each performed for 4 min with rest periods between the different speeds. Figure 1 shows the flow of the study design and framework.

### 2.3 Experimental equipment

#### 2.3.1 Triaxial accelerometer

**FIGURE 2:**
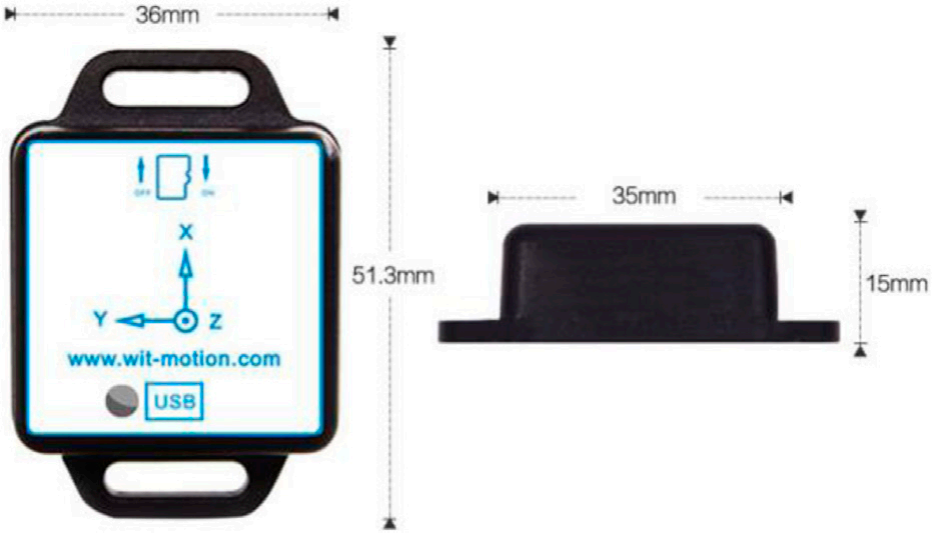
Accelerometer appearance.

The digital attitude sensor used in this study was the WT901SDCL model with storage function, manufactured by China Shenzhen Weite Intelligent Technology Co., Ltd., as shown in Figure 2. It had dimensions of 51.3 mm × 36 mm × 15 mm and a battery capacity of 200 mAh, and could sample at frequencies ranging from 0.1 Hz to 200 Hz with acceleration options of ±2/4/8/16 g. The module incorporated high-precision gyroscopes, accelerometers, and geomagnetic field sensors, and had a built-in rechargeable battery. Measured data were recorded on an SD card inside the module. The module supported a serial TTL interface for flexible connectivity options, and the serial port rate was adjustable from 2,400 bps to 921,600 bps. The accelerometer data were analyzed using the MiniIMU.exe software. Before the experiment, the sampling frequency was set to 100 Hz (Migueles et al., 2017), and the sensor was secured to the non-dominant wrist with a wrist strap.

#### 2.3.2 Quark PFT

In this study, the Quark PFT system (Cosmed, Italy) was used as the gold standard for measuring energy expenditure (METs). The Quark PFT is an advanced pulmonary function tester for gas-exchange analysis (VO_2_, VCO_2_). It ensures the accuracy and reliability of lung gas exchange and analysis, whether during exercise tests or rest periods, even during high-intensity exercise, using high-quality components and an ultrafast analyzer. The Quark PFT is a fixed system that utilizes both successive breathing and mixing chamber sampling technology, and has been scientifically verified for use in a wide range of exercise intensities. Before the experiment, the Quark PFT was calibrated using the flow sensor and standard gas, and participants’ information such as height, weight, and age were recorded on the Quark PFT for further analysis. The subjects wore the oxygen mask, and the tightness of the mask was adjusted and fixed with a head cap. After the mask was checked for airtightness, the data recording started.

### 2.4 Statistical analysis

The original acceleration data was preprocessed and the ANN model was established using Python 3.8. The relationship between acceleration data and METs was analyzed using SPSS 26.0, and the regression models for EE prediction were established using curve estimation. The accuracy of EE prediction under different models was compared by calculating the RMSE and Bias and measuring the consistency based on the Bland-Altman plot. The formulas used for calculating RMSE and Bias were as follows:

$$RMSE=\sqrt{\frac{\sum {\left({METs}_{forecast}-{METs}_{measured}\right)}^{2}}{N}}$$

$$Bias=\frac{\sum \left({METs}_{forecast}-{METs}_{measured}\right)/{METs}_{measured}}{N}.$$

## 3 Data preprocessing and model construction

### 3.1 Data preprocessing of multiple regression equations

The original acceleration data was preprocessed using Python 3.8. The processing of acceleration data includes three steps: Kalman filtering, correction gravity trend, and calculation of the vector magnitude (VM) of three-axis acceleration.

#### 3.1.1 Kalman filtering

The original acceleration data were processed using a Kalman filter, a versatile autoregressive filter often used for state estimation in dynamic multivariable systems. This filter is capable of estimating uncertain information and predicting the state at the next moment, even in the presence of noise interference, and can identify correlations between multiple variables that might otherwise be imperceptible. In this study, the data processed using the Kalman filter were less volatile and more stable.

#### 3.1.2 Correction of gravity trend

**FIGURE 3:**
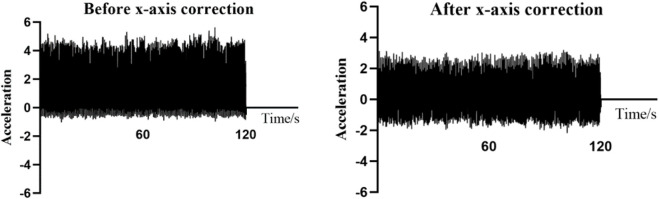
Regression equations expression of x-axis acceleration before and after trend removal.

In the experiment, we found that the accelerometer produced a small value even in the static state, which was attributed to the influence of gravity. Each axis was affected by the component of gravity. Following the suggestion of reference (Sekine et al., 2000), the acceleration data filtered by the three axes should be de-trended simultaneously. The formula is $X_{t}=X_{t}-X_{t-5∼t}$ . Figure 3 presents a comparison chart of the individual x-axis acceleration before and after the trend removal. Because the acceleration was generated in two directions, the processed acceleration in this study was closer to zero, which could effectively remove the gravity trend.

#### 3.1.3 Calculation of VM

Considering that wrist movement occurs in multiple axes, a single axis is insufficient to characterize the motion. Therefore, we combine the three-axis acceleration data into a composite acceleration called VM. The formula for calculating VM is: $\text{VM}=\sqrt{x^{2}+y^{2}+z^{2}}$ . The data synthesis method of the ActiGraph accelerometer is the integral value of the acceleration in every minute (i.e., counts/min). In this study, the mean value was used to replace the integral value (i.e., the mean value of the VM in 1 min, expressed as Mean). Corresponding Mean, and METs one by one to form multiple regression equations database.

### 3.2 Construction of regression equations

METs will increase with the increase in the Mean; however, when the Mean increases to a certain value, the METs will gradually slow down and conform to the characteristics of the logarithmic equation. Therefore, based on the established linear equation, we also established a logarithmic equation. In the regression equation, R^2^ represents the goodness of fit of the model. The larger the R^2^, the better the goodness of fit. SEE represents the standard error of the estimate of the model. The smaller the SEE, the better the prediction result of the regression equations. By comparing the R^2^ and SEE, the cubic equation with the larger R^2^ and SEE is selected from the established multiple regression equations. Therefore, a linear equation, logarithmic equation, and cubic equation were established in this study. The equation expressions are shown in Table 2.

**TABLE 2:** Expressions of regression equations.

Model	Name	Expression	R^2^	SEE
Model 1	Linear equation	METs = 8.33 Mean + 3.36	0.856	0.96
Model 2	Logarithmic equation	METs = 2.56 × ln (Mean) + 10.04	0.889	0.84
Model 3	Cubic equation	METs = 29.65 Mean^3^ − 52.67 Mean^2^ + 33.46 Mean + 1.22	0.891	0.83

Note: R^2^, R-Squared, commonly used to measure the fit of regression; SEE, standard error of estimate.

### 3.3 Construction of ANN model

#### 3.3.1 Selection of indicators

In this study, a feedforward ANN with a single hidden layer was adopted, which was mainly composed of an input layer, hidden layer, and output layer. In the process of establishing the input layer, there are multiple ways to extract input features and select window size. The input feature is obtained by first calculating the vector of magnitude (VM) of the original threeaxis acceleration data, and the features are then extracted from VM sequence with fixed window. The window is the fixed interval of the feature extraction. To determine the input features of the ANN model, the RMSE was compared under three windows (10 s, 30 s, 60 s), and 9 indicators were finally selected: the mean, sd, max, min, and 10th, 25th, 50th, 75th, and 90th percentiles of the VM sequence in the 60 s window. The number of neurons in the hidden layer was determined by adjusting the parameters, and the output was the predicted METs using ANN.

#### 3.3.2 Data normalization

To avoid unnecessary numerical computing and reduce the network training time, the input data are normalized: $x\_std=\left(x–x\_\min\right)/\left(x\_\max–x\_\min\right)$ *,* where x represents the original data, x_std represents the data after the normalization operation, and the values of x_std range from 0 to 1.

#### 3.3.3 Parameters of the model

The model parameters mainly include weight attenuation, the number of hidden neurons, and the number of iterations. In order to determine the optimal parameters, all the weights are attenuated between 0.1 and 0.9, and the number of hidden neurons is iterated between 1 and 30. The number of iterations is determined through model training. During the training process, when the number of iterations is 2,000, the model has converged. Finally, 2,000 iterations are used as the standard to stop the training to prevent overtraining. The weight attenuation and hidden neurons are adjusted using RMSE. When the weight attenuation is 0.8 and the number of hidden neurons is 8, the RMSE does not increase, the model achieves the optimal accuracy, and the ANN model is finally established (Model 4). Figure 5 shows the ANN model.

### 3.4 Construction of walking-and-running two-stage model

#### 3.4.1 Construction basis

**FIGURE 4:**
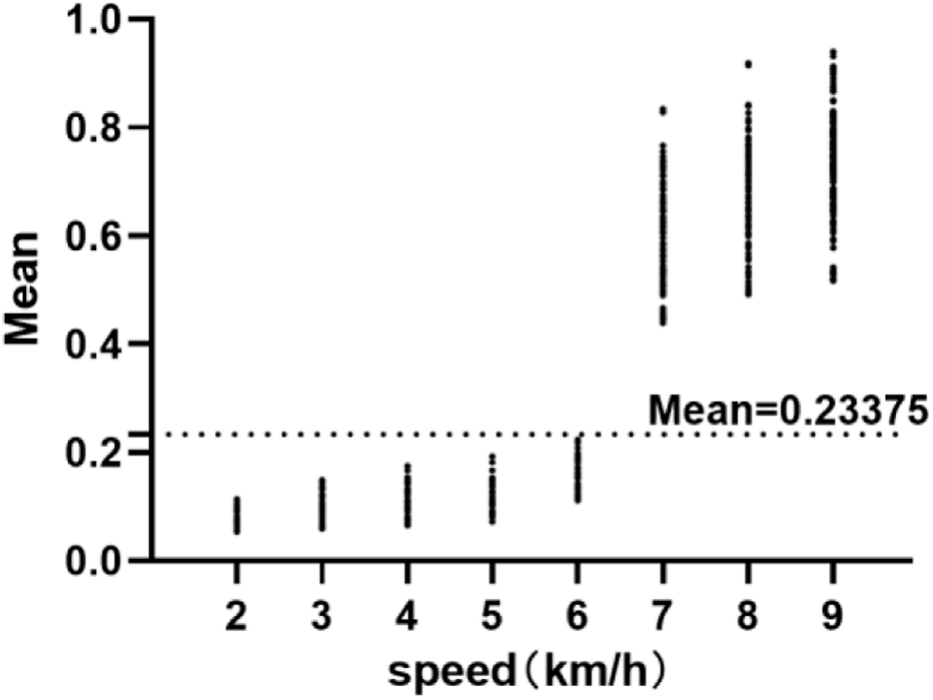
Mean scatter at different paces. Mean: Independent variables in linear regression equations.

This study developed separate EE prediction models for walking and running by modeling them separately. However, in order to build separate models for walking and running, it is necessary to have a good classification of these two activities. Through observation of the data, it was found that there was a large difference in the independent variable "Mean” between walking and running in the regression equation data preprocessing process. Figure 3 shows the scatter plot of Mean at various walking and running speeds. This study used the ROC curve method to classify Mean into two categories: walking and running. The ROC curve, also known as the “receiver operating characteristic curve,” is a curve that reflects the relationship between sensitivity and specificity. The entire graph is divided into two parts, and the area under the curve (AUC) of the part below the curve is used to indicate the prediction accuracy. The higher the AUC value, the higher the prediction accuracy. The maximum Youden index was used to find the cut-off point of Mean for walking and running, and the final cut-off point was determined to be Mean = 0.23375 (AUC = 1). Figure 4 shows the mean scatter at different paces. This cut-off point was then validated with a validation group, and the results showed that this method achieved 100% accuracy in classifying walking and running, laying the foundation for the development of the next two-stage model.

#### 3.4.2 Description of model

**FIGURE 5:**
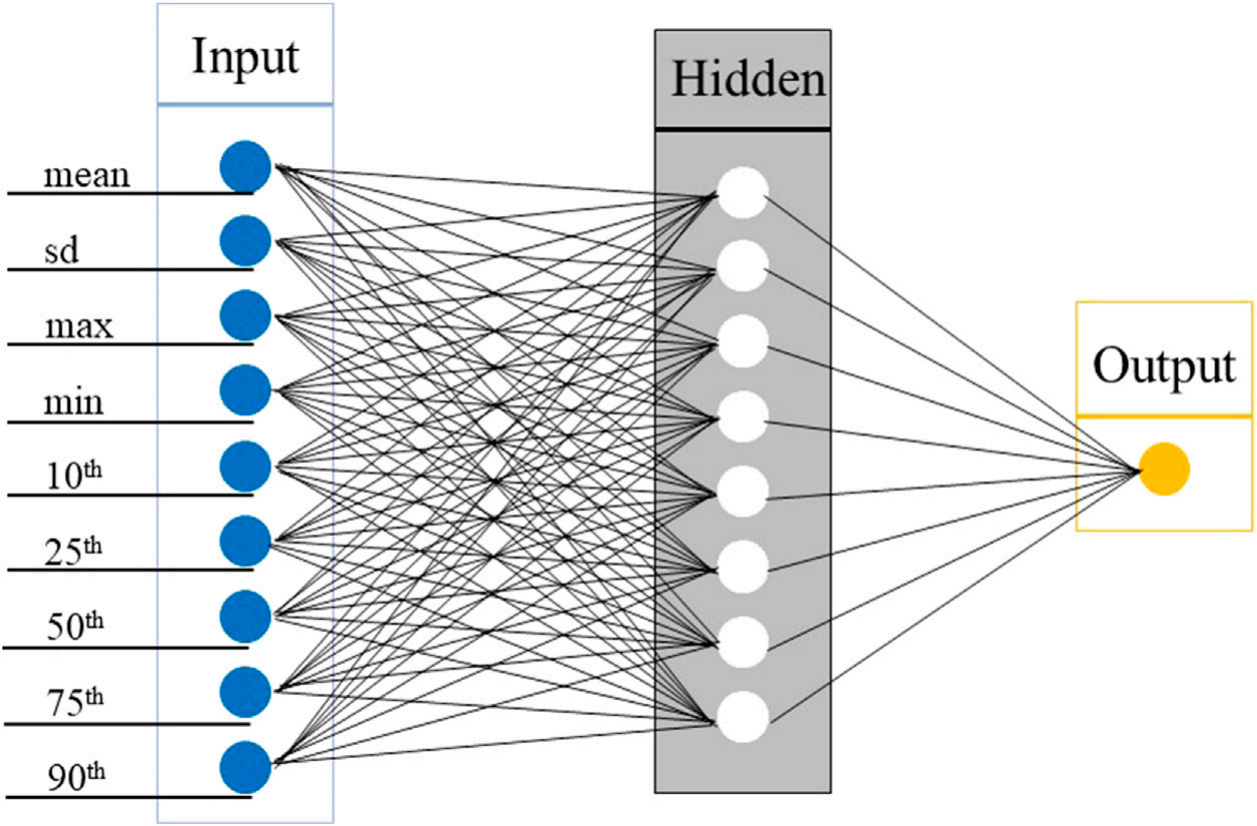
Diagram of two-stage model walking and running. ANN, Artificial Neural Network; VM, Vector Magnitude.

In this study, walking and running were separated. Based on the above modeling method of the ANN model, by adjusting the attenuation weight and number of hidden neurons, the ANN walking model achieved the best overall accuracy when the weight attenuation was 0.8 and the number of hidden neurons was 9. When the weight attenuation was 0.7 and the number of hidden neurons was 4, the ANN running model achieved the best overall accuracy. Finally, the ANN walking model and ANN running model were established separately from walking and running [that is, the walking-and-running two-stage model based on the ANN (Model 5)]. The model construction diagram is shown in Figure 5.

## 4 Result

### 4.1 Overall prediction error of models

**FIGURE 6:**
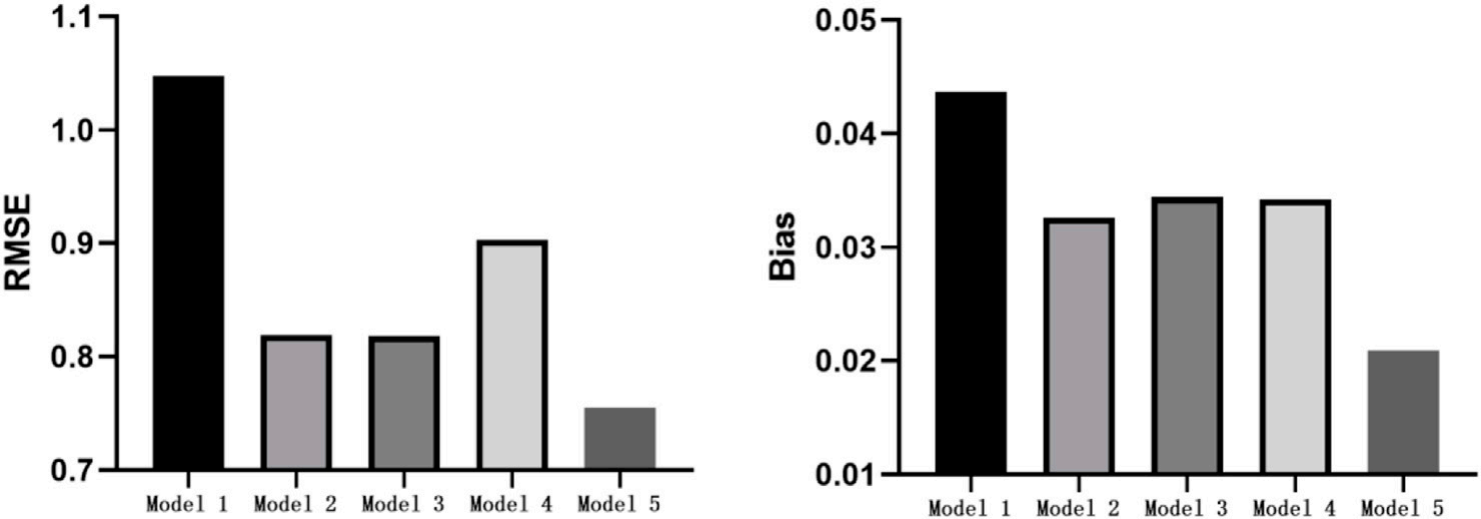
Overall RMSE and Bias comparison diagram of model. RMSE, Root Mean Square Error.

We compared the RMSE and Bias of EE prediction under five models. The smaller the RMSE and Bias values, the smaller the error between the predicted and measured METs. As shown in Figure 6, the RMSE and Bias under walking-and-running two-stage model were the smallest: 0.76 METs and 0.02 METs, respectively.

**FIGURE 7:**
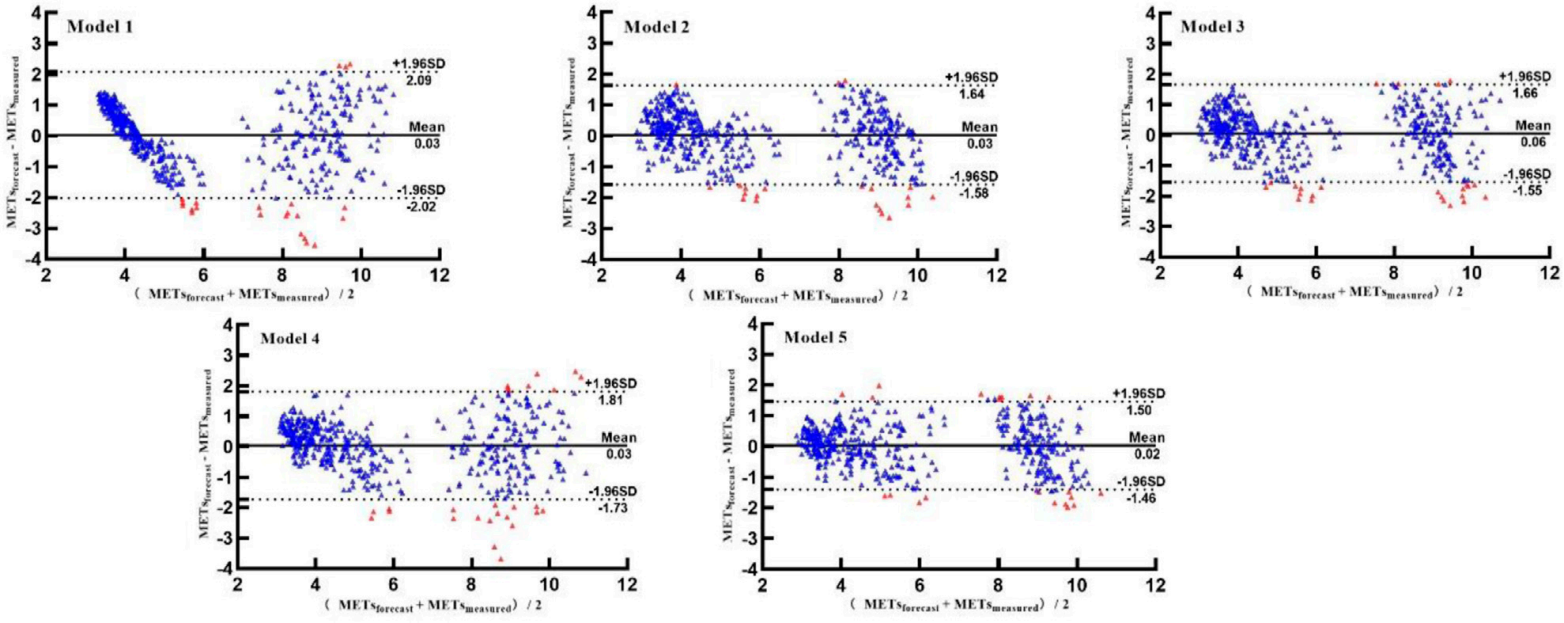
Bland–Altman plots of predicted and measured METs.

Based on the Bland–Altman plot, the consistency between the predicted METs of the five models and measured METs was further analyzed. As shown in Figure 7, the middle solid line represents the average value of the difference, and the two dotted lines represent the upper and lower lines of the 95% consistency limit. The fewer the points in the figure outside the dotted lines, the higher the consistency between the predicted and measured METs, and the more accurate the prediction. The five models have 22, 21, 24, 26, and 21 points outside the consistency interval, accounting for 4.58%, 4.38%, 5.00%, 5.42%, and 4.38% of the total points, respectively. The predicted METs of the logarithmic equation and walking-and-running two-stage model were highly consistent with the measured METs.

### 4.2 Prediction error of model for walking and running

**FIGURE 8:**
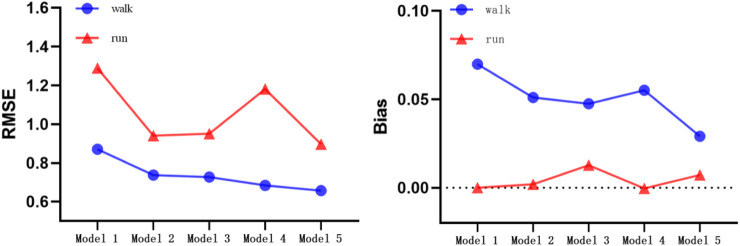
Comparison between RMSE and Bias for walking and running. RMSE: Root Mean Square Error.

Figure 8 shows the RMSE and Bias of walking and running under five models. By comparing the RMSE of walking and running, we found that the walking and running model can obtain the smallest error in walking (RMSE = 0.66 METs) and running (RMSE = 0.90 METs). By comparing the Bias of walking and running, the lowest Bias was obtained by walking-and-running two-stage model (Bias = 0.03 METs). In running, the linear equation, logarithmic equation, ANN model, and walking-andrunning model had lower Bias values (Bias <0.01 METs). In summary, the error of the walking and running in the two-step walking-and-running model was smaller than those of the other four models.

### 4.3 Prediction error of the models under different speeds

**FIGURE 9:**
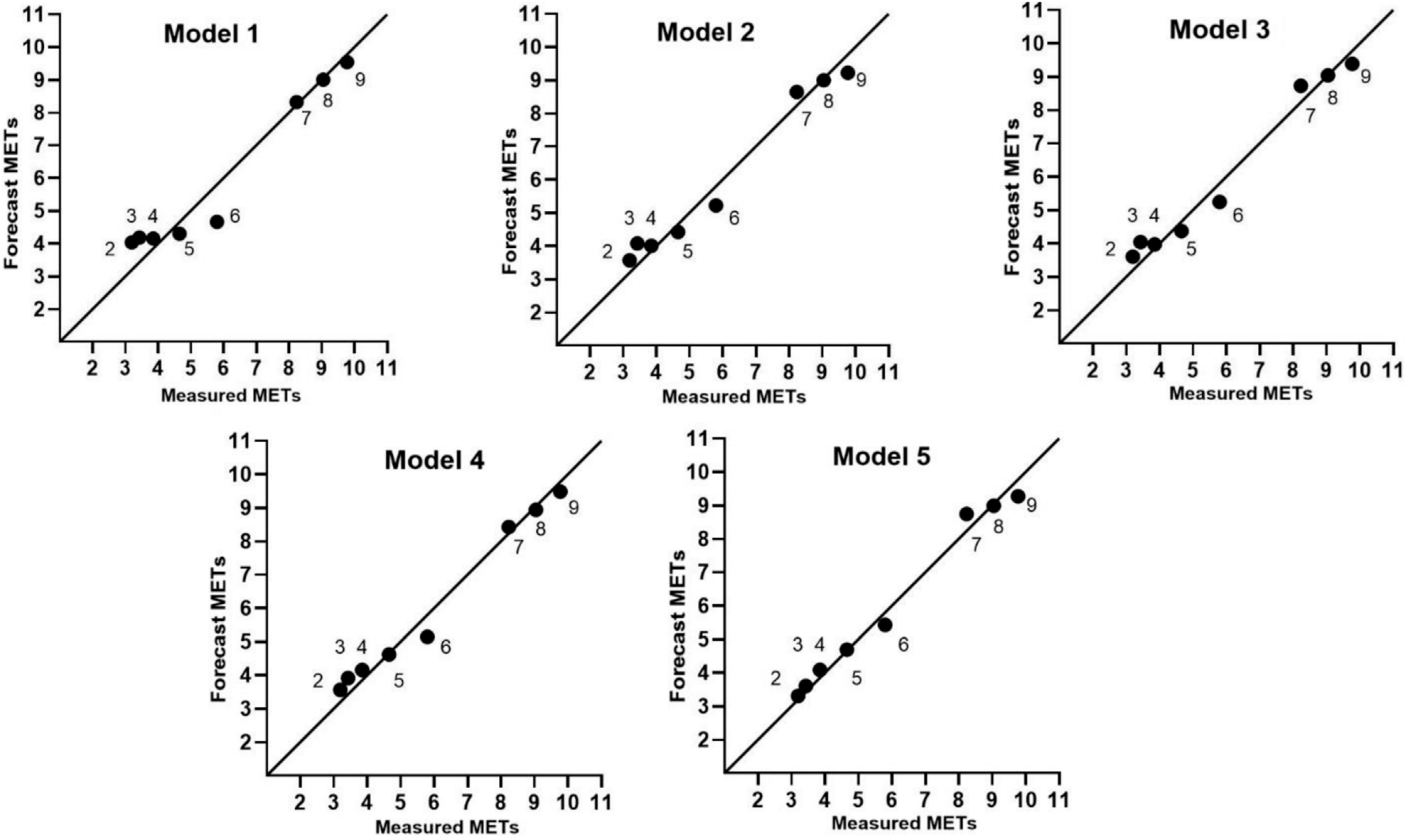
Mean value distribution of predicted METs and measured METs at various walking speeds. Measured METs, Average value of measured METs; Predicted METs, Predict the average value of METs.

Figure 9 shows the mean value of measured METs (*x*-axis) and predicted METs (y-axis). The reference line is y = x, and each point represents the mean value of the METs at different speeds. In the figure, the closer that the point is to the reference line, the smaller the prediction error of the model. The numbers of points on the reference lines of the linear equation, logarithmic equation, cubic equation, ANN model, and walking-and-running two-stage model were 3, 3, 3, 4, and 5, respectively. The walking-and-running two-stage model can obtain the maximum number of points on the reference line and the smallest error.

Next, we describe the comparison of the predicted METs and measured METs of five models at different walking/running speeds, by comparing the RMSE and Bias of the predictions. According to Tables 3, 4, the linear equation model had 3 speeds with prediction errors exceeding 10%, the logarithmic equation model had 2, the cubic equation model had 2, and the ANN model had 2. In contrast, the walking-and-running two-stage model had prediction errors below 10% for all speeds, with the highest error being 7%. Furthermore, the EE prediction error is within 5% for five different speeds. When comparing the RMSE for each speed, except for the speeds of 4 km/h and 5 km/h, the walking-and-running two-stage model had the lowest RMSE for the other 6 speeds. In summary, the walking-and-running two-stage model had better EE prediction accuracy than the other four models at different walking/running speeds.

**TABLE 3:** Error table of EE prediction under single step speed (RMSE).

Speed	Model 1	Model 2	Model 3	Model 4	Model 5
	RMSE	RMSE	RMSE	RMSE	RMSE
2	0.91	0.67	0.63	0.55	0.40
3	0.84	0.85	0.80	0.65	0.46
4	0.51	0.54	0.51	0.60	0.63
5	0.58	0.62	0.64	0.59	0.71
6	1.29	0.94	0.97	0.96	0.94
7	1.16	0.90	0.89	1.08	0.89
8	1.29	0.88	0.88	1.14	0.81
9	1.40	1.03	1.08	1.31	0.98

Note: EE, energy expenditure; RMSE, root mean square error.

**TABLE 4:** Error table of EE prediction under single step speed (Bias).

Speed	Model 1	Model 2	Model 3	Model 4	Model 5
	Bias	Bias	Bias	Bias	Bias
2	0.28 ± 0.13	0.13 ± 0.18	0.14 ± 0.16	0.13 ± 0.14	0.05 ± 0.12
3	0.23 ± 0.13	0.20 ± 0.17	0.19 ± 0.16	0.16 ± 0.13	0.06 ± 0.12
4	0.09 ± 0.11	0.05 ± 0.14	0.04 ± 0.13	0.09 ± 0.14	0.07 ± 0.15
5	−0.07 ± 0.09	−0.04 ± 0.12	−0.05 ± 0.12	0.00 ± 0.13	0.02 ± 0.16
6	−0.19 ± 0.09	−0.09 ± 0.12	−0.09 ± 0.13	−0.10 ± 0.12	−0.06 ± 0.13
7	0.02 ± 0.12	0.06 ± 0.10	0.07 ± 0.09	0.03 ± 0.13	0.07 ± 0.09
8	0.00 ± 0.14	0.00 ± 0.10	0.00 ± 0.10	0.00 ± 0.12	0.00 ± 0.09
9	−0.02 ± 0.14	−0.05 ± 0.09	−0.03 ± 0.10	−0.02 ± 0.13	−0.04 ± 0.08

## 5 Discussion

The accuracy of the EE prediction of the model was verified through the validation group data. The results showed that the walking-and-running two-stage model established by dividing walking and running into two stages exhibited good EE prediction accuracy, whether in overall prediction, walking and running separately, or at various walking speeds. The walking-and-running two-stage model performed better than other models established in this study. This can also indicate that establishing a single exercise energy consumption algorithm for physical activity, and then comparing physical activity with its energy consumption algorithm through classification, can effectively improve the accuracy of energy consumption prediction for physical activity. The improvement of energy consumption prediction accuracy is conducive to more accurate monitoring of sports energy consumption, selecting appropriate exercise measures based on one’s own situation, participating in sports more scientifically, and promoting physical health.

### 5.1 Accuracy analysis of model EE prediction

The linear equation showed the lowest accuracy in predicting EE among all the models. By wearing accelerometers on both wrists, Montoy compared the linear equation with the ANN model. The results showed that the two linear equations had lower correlations and higher RMSE than the ANN model (Montoye et al., 2017). Although the initial model established in the field of accelerationbased energy consumption prediction was a linear model, with the continuous progress of research and the constant changes in the wearing position, the linear equation established by the wrist-worn accelerometer has significant errors in predicting EE. The applicability of the linear equation in wrist model construction is poor.

The accuracy of logarithmic and cubic equations for EE prediction is better than that of linear equation, showing lower RMSE and Bias in overall prediction errors, and good performance in consistency measurements. The logarithmic equation performs better than the ANN model in all three indicators, indicating good prediction accuracy. Combining the validation results of walking and running separately and at various speeds, the logarithmic equation has higher EE prediction accuracy and more stable performance, especially during the running phase, with RMSE lower than that of the cubic equation and the ANN model, and Bias less than 0.001 METs. Considering that the oxygen uptake gradually increases to a maximum and then remains constant during exercise, the accuracy of EE prediction during running by the logarithmic equation is affirmed. When evaluating the cubic equation, according to its graph and expression, as the speed increases, the Mean value gradually increases, and METs gradually tends to be stable. However, when the Mean is greater than 0.59, the energy consumption gradually increases again, and the rate of increase gradually becomes larger. Therefore, although the cubic equation exhibits good energy consumption prediction accuracy in this study, caution should be exercised when using it in practical applications.

The difference between this study and the research of other scholars is that the overall prediction accuracy of the ANN model built in this study was 0.90 METs, which was not lower than the logarithmic equation and cubic equation. The excessive noise in the original data may have had a certain impact on the modeling. In this study, we also established the ANN model through the data after noise reduction and trend removal in the regression equation; however, the results show that the RMSE did not decrease. Staudenmayer established an ANN model and verified the models of other scholars, indicating that the ANN model is better than the equation model in EE prediction (Staudenmayer et al., 2009). In this study, the accuracy of the ANN model in predicting the walking EE was higher than those of the logarithmic equation and cubic equation in the verification of the accuracy of the walking–running EE prediction. At the same time, based on the ANN model, the accuracy of the EE prediction is the best in all models through the walking-and-running two-phase model established by separating the walking and running.

### 5.2 Analysis of model construction mode

#### 5.2.1 ANN model input-layer index selection

The construction of the ANN model was based on the original acceleration and MET data. The nine features of the input layer were determined through a literature review; however, this does not mean that the accuracy of the model could not be improved after the inclusion or replacement of other features. Rothney extracted the median, skewness, kurtosis, and other features from the original data in the input layer, and the built model had good prediction accuracy (Mackintosh et al., 2016). Ruch constructed the model by incorporating the acceleration count, VM, steps, and inclination of the three axes, and it also had a good prediction performance (Ruch et al., 2013). However, the selection of features in this study was performed by summarizing the methods of feature extraction in most studies. The mean value reflects the concentration trend of the dataset, the standard deviation reflects the dispersion degree of the dataset, the maximum value and minimum value reflect the extreme values of the dataset, the percentile is used to measure the data position, the 25th, 50th, and 75th are commonly used in the description of the box diagram, and the 10th and 90th refer to the low and high values in unit time in a stable state during signal processing, respectively. In this study, the ANN model was established by incorporating nine features. The results showed that the ANN model was more accurate in predicting the walking EE, and that the accuracy of the walking-and-running two-stage model was better than those of the other models built in this study.

#### 5.2.2 Establishment of EE model with walking speed as independent variable

In the experiment, Brooks established an EE prediction model based on wearing hip-mounted CSV accelerometers and walking on horizontal terrain. The results showed that the speed-based energy consumption model was more accurate than the CSA-based energy consumption model (Brooks et al., 2005). In this study, we also attempted to construct the model based on the acceleration data and adding walking speed as an independent variable. The results showed that adding walking speed as an independent variable improved the accuracy of both the regression equation and the ANN model for predicting energy consumption, and was lower than the walking-and-running two-stage model constructed in this study. However, considering that in practical applications, this model is only limited to predicting EE on a treadmill with known walking speed, but requires accurate prediction of walking speed in a free state. This study also attempted to use 9 features of ANN as input variables and walking speed as output variable to predict walking speed using an ANN model, but did not achieve satisfactory results. In addition, walking speed can be predicted by a mobile phone or a sports bracelet, and this study used multiple mobile apps to conduct experiments on a treadmill, but found that the predicted speed had a certain deviation from the actual speed of the treadmill, especially at faster or slower speeds.

Therefore, this study did not construct a model with walking speed as an independent variable, but adding walking speed as an independent variable can improve the accuracy of EE prediction for various walking speeds.

#### 5.2.3 Selection of window size of ANN model

Trost points out that the ANN window size has a certain impact on the accuracy of the EE prediction. The window size increases from 10 s to 60 s, the Bias decreases from 0.3 METs to 0.2 METs, and the RMSE decreases from 1.1 METs to 0.9 METs, which is the most significant in a variety of physical activities (Trost et al., 2012). As the window size increases from 10 s to 60 s, the Bias decreases from 0.6 METs to 0.2 METs, and the RMSE decreases from 1.1 METs to 0.7 METs (Trost et al., 2012). In the selection of the model window, the index is selected through the 60 s window. In addition, the 10 s and 30 s windows are also tried to select the index. According to the results of the correlation analysis, the correlation between the Mean and METs under the 10 s and 30 s windows was reduced, and the correlation under the 10 s window was the lowest. ANN models are established based on 10 s and 30 s windows, and the RMSE under different windows are obtained. The results show that the RMSE under the 10 s (RMSE = 0.99 METs) and 30 s (RMSE = 0.93 METs) windows were bigger than the RMSE under the 60 s (RMSE = 0.90 METs) window, which is consistent with Trost’s experimental results (Trost et al., 2012). To improve the accuracy of the EE prediction in various speeds, the 60 s window was selected.

### 5.3 Limitations

The subjects of this study were college students aged 18–30 years, and the effectiveness in other age groups has not yet been verified. The experiment was carried out on a treadmill, which is limited by the experimental environment and conditions and lacks the verification of asynchronous speed and EE in real life. In this study, we only conducted the establishment of the EE prediction model for walking and running and did not conduct experiments for other sports. In future experiments, we will further establish EE prediction models for other sports.

### 5.4 Future outlook

In addition, the walking-and-running two-stage model was the optimal model in this study; however, its calculation process is relatively complex, and to obtain the accurate classification of walking and running, a large number of computations need to be implemented. However, in future research, we hope to classify physical activity, establish the EE prediction models of various physical activity types, and create an EE algorithm corresponding to physical activity that will be called the physical-activity-type recognition algorithm, which can be used as a method to improve the accuracy of model EE prediction.

#### 5.4.1 EE prediction algorithm based on physical-activity-type recognition

In recent years, with the continuous development of artificial intelligence, PA-type recognition based on accelerometers has become a research hotspot. In future research, through the EE prediction experiment of a single sports event, we hope to establish the corresponding EE prediction model, and to establish an algorithm library of EE prediction models through multiple sports. In application, different sports events can be classified through the PA-type recognition algorithm, and then different EE prediction models can be established, constantly improving the accuracy of the EE prediction.

#### 5.4.2 Monitoring motion load based on acceleration data

The monitoring of the external loads of athletes using accelerometers is simple, noninvasive, and the operation is easy. Based on the prediction model of the EE of a single sports item, it can be applied in sports training to monitor the exercise load and to establish a scientific exercise load evaluation system to achieve a reasonable combination of training and rest and reduce the occurrence of sports injuries.

## 6 Conclusion

Through the processing of original data, the establishment of various models, and the verification of the accuracies of the EE predictions of the models, we found that the construction method of the EE prediction model affects the accuracy of the three-axis accelerometer in the estimation of the EE of human motion. In estimating the accuracy of walking and running EE prediction, the ANN-based walking-and-running two-stage model established by separating walking and running is superior to the other models built in this study, can better estimate the walking and running EE. The improvement of energy consumption prediction accuracy is conducive to more accurate monitoring of sports energy consumption, selecting appropriate exercise measures based on one’s own situation, participating in sports more scientifically, and promoting physical health.

## Data Availability

The original contributions presented in the study are included in the article/Supplementary material, further inquiries can be directed to the corresponding author.

## Footnotes

1 The original data is available at https://figshare.com/projects/Raw_data_of_accelerometer_and_MET_values/178782 and the code is available at https://github.com/zjxcode666/Predicting-Energy-Expenditure-Based-on-a-Wristwear-Three-axis-Accelerometer.
